# Photosynthetic Microorganisms in Plant Growth Promotion and Stress Response: Proposed Organisms with In Silico Validation

**DOI:** 10.3390/plants15111634

**Published:** 2026-05-26

**Authors:** Olga Dimitra Asvesta, Eleni Kotsadam, Evangelia Mouchtaropoulou, Anagnostis Argiriou

**Affiliations:** Department of Food Science and Nutrition, University of the Aegean, 81400 Myrina, Lemnos, Greece; oasvest@bio.auth.gr (O.D.A.); kotsadam.elen@gmail.com (E.K.); eva.mouchtaropoulou@certh.gr (E.M.)

**Keywords:** microalgae, cyanobacteria, biostimulants, biofertilizers, abiotic stress tolerance, sustainable horticulture

## Abstract

The transition towards sustainable agri-food systems necessitates the development of effective and technologically advanced biofertilizers and biostimulants capable of reducing reliance on synthetic agrochemicals while enhancing crop productivity. Photosynthetic microorganisms, including cyanobacteria and microalgae, represent promising biological platforms owing to their extensive metabolic potential, their ability to synthesize high-value bioactive compounds, and, in certain cases, their capacity for atmospheric nitrogen fixation. These properties make them particularly valuable for enhancing plant growth and improving tolerance to abiotic and biotic stresses. In this study, a systematic review was conducted to identify diverse cyanobacterial and microalgal taxa with demonstrated roles in plant growth promotion and stress mitigation through multiple mechanisms and adaptive traits. A subset of these microorganisms was subsequently curated into a targeted database and subjected to bioinformatics analyses, leading to the identification of key metabolic pathways associated with stress response and plant growth promotion.

## 1. Introduction

The modern agricultural sector faces multiple and often competing challenges, including the need to increase crop productivity, ensure global food security, and reduce the environmental impacts associated with the extensive use of synthetic fertilizers and agrochemicals. The intensive application of these inputs has been strongly linked to soil degradation, eutrophication of aquatic ecosystems, and increased greenhouse gas emissions, highlighting the urgent need for sustainable and environmentally friendly alternatives [1,2]. Moreover, the continued reliance on synthetic agrochemicals raises concerns about the accumulation of residues in agricultural products, potential health risks for humans, and the disruption of soil microbiomes and ecosystem biodiversity, all of which are increasingly scrutinized by regulators and consumers [3,4].

In this context, biofertilizers and biostimulants have emerged as promising strategies to support sustainable agriculture. Biofertilizers are formulations of living microorganisms that enhance the availability of essential nutrients to plants, while biostimulants are substances or microorganisms that improve plant growth, nutrient use efficiency, and resilience to abiotic and biotic stress without directly supplying nutrients [5,6,7]. Biostimulants include a wide range of materials, such as humic and fulvic acids, protein hydrolysates, seaweed extracts, vitamins, and plant-growth-promoting microorganisms. These agents function via diverse physiological and molecular mechanisms, including modulation of hormonal pathways, stimulation of antioxidant systems, and modulation of soil microbiota, often independently of traditional nutrient provision [8,9,10].

Among biostimulant and biofertilizer sources, photosynthetic microorganisms, including cyanobacteria and microalgae, have garnered particular attention due to their exceptional metabolic versatility and biotechnological potential. These organisms are capable of producing a variety of bioactive compounds, such as carotenoids, phycobiliproteins (e.g., phycocyanin), polysaccharides, and phytohormones, which have been demonstrated to enhance seed germination, root and shoot development, nutrient uptake, and overall plant physiological performance [11,12]. Additionally, certain cyanobacteria possess the ability to fix atmospheric nitrogen, converting it into forms available to plants, thereby reducing the need for inorganic nitrogen fertilizers and contributing to soil fertility, particularly in organic and low-input farming systems [13]. Their capacity to sequester carbon dioxide further enhances their environmental value, presenting opportunities for climate-smart agricultural applications.

Despite their potential, the large-scale adoption of photosynthetic microorganisms in agriculture remains limited. Variability in outcomes between studies has led to diverging views on their efficacy, especially under different environmental conditions, crop species, and soil types. Challenges include the targeted selection of strains with reliable functional traits, the optimization of cultivation and formulation processes to maximize biomass and metabolite yields, and the standardization of application methods to ensure reproducible effects in the field [14]. Furthermore, although in vitro and greenhouse studies often demonstrate promising results, translating these benefits into large-scale field applications is not straightforward, and some studies report inconsistent or context-dependent outcomes, highlighting the need for systematic evaluation.

Recent advances in bioinformatics and in silico approaches offer powerful tools to address these knowledge gaps. High-throughput omics technologies, including metagenomics, transcriptomics, metabolomics, and comparative genomics, enable the identification of genes, biosynthetic pathways, and regulatory networks associated with plant growth promotion and stress tolerance. Computational approaches such as genome annotation, functional enrichment analysis, metabolic pathway reconstruction, protein–protein interaction networks, and machine learning models can be used to predict microbial functional traits and plant–microbe interactions. In addition, multi-omics integration facilitates the identification of key biomarkers linked to nutrient acquisition, phytohormone production, and abiotic stress resilience. These approaches support the rational selection of beneficial microbial strains and the development of more efficient biofertilizer and biostimulant formulations, thereby helping bridge the gap between laboratory discoveries and practical agricultural applications.

Against this background, the present review aims to provide a comprehensive overview of photosynthetic microorganisms as sources of biofertilizers and biostimulants, focusing on their mechanisms of action in promoting plant growth and mitigating abiotic and biotic stresses. By integrating evidence from the literature with in silico analyses, this work identifies key metabolic pathways and bioactive compounds associated with these beneficial effects. Taken together, this review highlights the dual role of photosynthetic microorganisms as biostimulants and biofertilizers and emphasizes how in silico approaches can support the identification of metabolic pathways that enhance plant growth and stress resilience, offering a roadmap for sustainable and climate-smart agricultural practices.

## 2. Photosynthetic Microorganisms & Optimal Growing Conditions

A wide range of products has been developed to enhance crop productivity. However, it is important to recognize that the categories of biofertilizers, biostimulants, and other biologically derived agricultural inputs often overlap in their functions and mechanisms of action, as acknowledged in current regulatory and scientific frameworks. According to the European Union Fertilising Products Regulation (EU 2019/1009) [7], plant biostimulants are defined by their ability to stimulate plant nutrition processes independently of nutrient content, thereby improving nutrient use efficiency, stress tolerance, crop quality, or nutrient availability in the rhizosphere. In practice, many biological products exert multiple and complementary effects simultaneously, including:

(i) improving soil physicochemical and biological properties, thereby enhancing nutrient availability and soil fertility;
(ii) protecting crops against biotic and abiotic stressors, including pathogens, pests, drought, salinity, and temperature extremes;
(iii) directly stimulating plant growth and development through modulation of physiological and metabolic processes.

In this context, products derived from microalgae and cyanobacteria, as well as their biomass, have attracted increasing attention as sustainable and multifunctional inputs in modern agriculture. Depending on their composition and mode of application, these products can be broadly classified as biofertilizers, biostimulants, and bioinsecticides. Biofertilizers primarily enhance nutrient availability through mechanisms such as nitrogen fixation, phosphorus solubilization, and improvement of soil microbial activity. In addition, microalgal–nitrogen-fixing bacterial consortia have been reported to enhance plant growth through synergistic effects on nutrient cycling and phytohormone production, representing a promising approach for sustainable agriculture [13,15]. A well-characterized example is the co-culture of *Chlorella vulgaris* with *Azospirillum brasilense*, in which the bacterial partner supplies fixed nitrogen and indole-3-acetic acid (IAA) while benefiting from algal-derived oxygen and organic carbon, resulting in increased biomass and improved plant growth-promoting activity compared with monocultures [16]. Biostimulants, on the other hand, promote plant growth and resilience by modulating physiological processes, including hormone-like activity, improved nutrient uptake, and enhanced stress tolerance. Bioinsecticides contribute to crop protection by suppressing or controlling pest populations through natural bioactive compounds or microbial activity, offering an environmentally friendly alternative to conventional chemical pesticides.

To compare the agricultural relevance of the selected photosynthetic microorganisms, we used a literature-based qualitative assessment aligned with the horticultural scope of this review. Two criteria were considered. The first was the reported ability of each organism to produce bioactive compound classes associated with plant growth promotion and stress mitigation, namely phenolic compounds, carotenoids, terpenoids, polysaccharides, free fatty acids, and phytohormones. These compound classes were not prioritized or weighted differentially; all were treated equally in the assessment. The second criterion was tolerance to cultivation-relevant environmental parameters, used here as a proxy for biological robustness and adaptability. These parameters included light intensity and photoperiod (light/dark cycle), temperature, pH, nutrient availability, and CO_2_ concentration, all of which influence growth performance, metabolic activity, and overall biomass productivity.

### 2.1. Plant Growth Promotion

The direct stimulation of plant growth is mainly facilitated by primary and secondary metabolites that enhance the physiological state of the crop and the biological health of the soil. A critical class of these compounds is polysaccharides, which are synthesized by genera such as *Dunaliella*, *Chlorella*, *Navicula*, and *Nostoc*. These molecules function as elicitors of plant defense and stress-response pathways while simultaneously improving soil quality and stimulating plant development [17,18,19]. Their multi-functional role makes them indispensable for biofertilization strategies aimed at increasing yields through improved soil structure and nutrient availability [20].

In addition to carbohydrates, carotenoids synthesized by species like *Arthrospira* sp., *Dunaliella salina*, and *Haematococcus pluvialis* contribute significantly to crop enhancement. These pigments provide antioxidant protection and facilitate biological soil restoration and fertilization. Furthermore, phytohormones and terpenoids, identified in organisms such as *Chlamydomonas*, *Arthrospira*, and *Synechocystis* sp., act as chemical messengers that regulate cellular activities and attract pollinators. These compounds directly stimulate growth and development, ensuring that the plant can maximize its genetic potential under various field conditions.

The efficacy of these growth-promoting agents is inextricably linked to achieving high biomass productivity, which is heavily influenced by environmental factors such as temperature and lighting [21]. Research indicates that for *Chlorella* and *Scenedesmus* species, the highest biomass yields in tubular photobioreactors are achieved at temperatures of 29–32 °C and a pH of approximately 8.4 [22]. Furthermore, species like *Selenastrum minutum* have demonstrated exceptionally high growth rates, reaching 1.73 d^−1^ under specific laboratory conditions of 35 °C and 420 μmol m^−2^ s^−1^ irradiance [21]. This suggests that identifying the precise environmental thresholds for each strain is vital for the commercial viability of growth-promoting products.

Finally, the role of carbon availability cannot be overlooked, as biomass production is directly correlated with CO_2_ absorption [21]. In cultures of *Scenedesmaceae* (specifically the EZB1 strain), the application of 2% CO_2_ combined with a high photon flux of 112.32 mol m^−2^ day^−1^ has been shown to maximize growth rates and biomass production [23]. Under these conditions, a stable mass-based ratio between CO_2_ absorbed and dry biomass produced (MCO_2_/Mbiomass) of approximately 2.0 has been reported, with comparable values (1.8–2.3) consistently observed across different microalgal species. This ratio reflects the carbon content of microalgal biomass, which is approximately 50% by mass, corresponding to a theoretical stoichiometric minimum of ~1.83 g CO_2_ per g biomass; empirical values close to 2.0 therefore indicate efficient channeling of fixed carbon into biomass, with limited losses through respiration or release of inorganic carbon [21].

### 2.2. Stress Response Adaptation

Photosynthetic organisms are equally valued for their ability to produce metabolites that protect crops and enhance their resilience against biotic and abiotic stressors, including pathogens, pests, and environmental extremes [20]. Phenolic compounds, identified in species like *Chlorella vulgaris*, *Isochrysis* sp., and *Phaeodactylum tricornutum*, provide robust antioxidant, antibacterial, and antifungal defenses [20]. These substances shield crops from pathogens and adverse conditions by neutralizing reactive oxygen species and inhibiting the growth of harmful microorganisms [20]. According to Madja et al. [24], the maximum accumulation of these phenolic compounds in *Chlorella* is often triggered by controlled environmental stress rather than optimal growth conditions.

This stress-induced synthesis is particularly effective when high light intensity is combined with limited nitrogen availability, activating the organism’s antioxidant defense mechanisms [24]. The timing of the harvest is a decisive factor in securing these protective metabolites, as the peak concentration of phenolics is generally observed at the end of the exponential or the beginning of the stationary growth phase [24]. Similarly, free fatty acids from genera such as *Nannochloropsis*, *Anabaena*, and *Scenedesmus* provide antioxidant, antiviral, and antibiotic protection [20]. The specific profile of these lipids, such as polyunsaturated fatty acids (PUFAs) and ω-3 linolenic acid, can be significantly altered by adjusting light intensity and the nitrogen/carbon supply [25].

The robustness and adaptability of the selected strains are critical for ensuring stable production of defense-related compounds across different environments [21]. For instance, *Dunaliella salina* is recognized for its high resistance to environmental fluctuations, while *Arthrospira platensis* thrives in thermophilic conditions between 30–35 °C [21]. Conversely, species like *Phaeodactylum tricornutum* are adapted to cooler temperatures, with growth being inhibited above 25 °C [21]. This diversity in environmental tolerance allows for the selection of specific organisms tailored to the climatic requirements of the intended agricultural application [21].

The choice of cultivation system also influences the productivity and consistency of stress-response products [26]. Open systems are widely used due to their relatively low operational cost and scalability, although they are more susceptible to contamination and environmental fluctuations, which can reduce productivity [27]. In contrast, closed photobioreactors minimize contamination risks and allow better control of cultivation parameters such as light, temperature, and CO_2_ supply [28]. Although associated with higher capital and operational costs, closed systems have been reported to achieve higher biomass productivities under optimized conditions, often in the range of 1.5–3-fold compared to open systems, depending on species and operational setup [27,29]. Consequently, they can improve the consistency and purity of bioactive compounds relevant for crop protection.

Table 1 summarizes the bioactive compounds collectively reported in the literature, including their biological functions and the microbial strains in which they are most abundantly produced.

## 3. Assessment of Biostimulant Activity

The literature review indicates that the bioactivity of strains can be effectively assessed using a range of functional indicators, including:

- Nitrogen-fixing capacity;
- Carotenoid production;
- Phycocyanin production;
- Production of amino acids and antioxidant compounds.

These indicators serve as key proxies for evaluating the metabolic potential and functional relevance of the strains, especially when concerning horticulture. In particular, they provide valuable insight into mechanisms through which these microorganisms may promote plant growth, enhance stress tolerance, and improve overall plant health.

Moreover, such parameters offer a practical framework for the preliminary screening and selection of promising strains prior to in vivo or field-level experimentation. By focusing on these bioactivity markers, future studies can be more strategically designed to investigate the specific effects of the tested formulations on target plant species under varying environmental conditions.

Overall, the integration of these biochemical and physiological indicators lays the foundation for targeted experimental validation, supporting the development of efficient and sustainable applications of these formulations in agricultural systems.

### 3.1. Effects of Microalgae and Cyanobacteria Applications on Crop Protection

The application of microalgae- and macroalgae-derived products has been widely reported to enhance plant growth, productivity, and nutritional quality across a variety of crops. For instance, aqueous extracts of *Gracilaria corticata* and *Enteromorpha flexuosa* increased shoot and root length, dry biomass, and nutritional value in maize (*Zea mays* L.) and sunflower (*Helianthus annuus* L.), as reflected by higher levels of photosynthetic pigments, carbohydrates, proteins, and essential nutrients [30]. However, the effectiveness of algal-derived biostimulants can vary considerably depending on algal species, extraction method, application dose, crop type, environmental conditions, and cultivation practices. In some cases, neutral or inconsistent responses have also been reported, highlighting the need for standardized formulations and field-scale validation studies [31,32,33].

Similarly, the application of dry biomass from *Chlorella vulgaris* and *Arthrospira platensis* in maize cultivation improved germination rates and significantly enhanced plant yield. Increases were also observed in shoot length, leaf number, and both fresh and dry weights of roots, shoots, and whole plants [34]. In sweet corn (*Zea mays* var. *saccharata*) and spinach (*Spinacia oleracea*), *Chlorella minutissima* biomass enriched soil nutrient content and promoted plant growth [35].

In tomato (*Solanum lycopersicum*) cultivation, extracts of *Chlorella vulgaris* improved plant growth and yield while increasing the availability of macro- and micronutrients in the soil [36]. Furthermore, crude polysaccharide extracts from *Arthrospira platensis*, *Dunaliella salina*, and *Porphyridium* sp. enhanced plant growth, increased node number, and elevated levels of carotenoids, chlorophyll, and proteins, alongside boosting the activity of key enzymes such as nitrate reductase and NAD-glutamate dehydrogenase [37].

The use *of Monoraphidium* sp. biomass in tomato cultivation reduced nitrate leaching from the soil, while maintaining plant growth comparable to that achieved with synthetic fertilizers [38]. Likewise, polysaccharide extracts from *Arthrospira platensis* improved plant growth, root biomass, and node development in tomato and pepper (*Capsicum annuum*) crops. Enhanced shoot and root development in tomato and cucumber (*Cucumis sativus*) has also been reported following treatment with *Chlorella vulgaris* [39,40]. In addition, a combination of *Chlorella vulgaris* and *Chlorella sorokiniana* was associated with increased activity of defense-related enzymes in tomato plants [17].

Further evidence highlights synergistic effects when microalgae are combined with other organic inputs. The application of *Arthrospira platensis* alongside dried cattle manure improved growth, yield, and leaf pigment content in onion crops [41]. Consistently, biofertilizers derived from *Scenedesmus quadricauda* enhanced shoot and root growth in lettuce (*Lactuca sativa*) and activated enzymes related to secondary metabolism [42]. Moreover, the combined use of *A. platensis* and *C. vulgaris* resulted in greater improvements in shoot length and leaf number—by 37% and 40%, respectively—compared to individual applications, indicating synergistic interactions [34]. Notably, *A. platensis*-based biofertilizers significantly increased shoot height in bean (Phaseolus vulgaris) within 50 days, while *C. vulgaris* exhibited measurable effects within just 20 days [34].

In another study, *Tetraselmis* sp. biomass applied as a biofertilizer in date palm (Phoenix dactylifera) cultivation led to increased plant growth rates, enhanced rooting capacity, greater leaf number, longer shoots, and higher total chlorophyll content compared to conventional fertilizers. Soil analysis further revealed increased levels of nitrogen, potassium, and phosphorus, highlighting improved soil fertility [43].

The beneficial effects of *Arthrospira platensis* biomass were also demonstrated in crops such as *Eruca sativa*, *Amaranthus gangeticus*, *Brassica rapa* ssp. *chinensis*, and *Brassica oleracea alboglabra*, where it enhanced plant growth, improved germination, and increased yield and grain weight [44].

Additionally, biofilms composed of cyanobacteria and fungi, specifically *Anabaena torulosa* and *Trichoderma viride*, improved seedling traits, enzymatic activity, and nutrient mobilization at the seed stage in maize inbred genotypes V6 (HKI323PV) and V7 (HKI161PV) [35].

Finally, microbial consortia including *Chlorella*, *Anabaena*, *Scenedesmus*, *Chroococcus*, *Chlorococcum*, *Fischerella*, *Phormidium*, *Westiellopsis*, and *Spirogyra* have been shown to enhance wheat (*Triticum aestivum* L. HD2967) cultivation. Their application increased the availability of macronutrients, improved plant dry biomass, and boosted crop productivity and yield, while also stimulating overall microbial activity in the soil [45].

Collectively, these findings highlight the significant potential of microalgae- and cyanobacteria-based biofertilizers as sustainable alternatives to conventional agrochemicals, contributing to improved plant performance, soil fertility, and agricultural productivity.

### 3.2. Effects of Microalgae and Cyanobacteria Applications on Tree Crops

The demand for tree seedlings has increased in recent years as a result of large-scale afforestation and reforestation initiatives aimed at expanding forest cover. To investigate whether algae extracts can enhance plant development, Comin et al. [46] conducted an experiment at the ERSAF Regional Forest Nursery in Curno, Italy. Seeds of five woody species (*Amelanchier ovalis*, *Crataegus monogyna*, *Carpinus betulus*, *Fagus sylvatica*, and *Ligustrum vulgare*) were sown in trays containing substrates enriched with 0×, 1×, 2×, or 3× the recommended dose of a pure *Ascophyllum nodosum* extract. Following germination, 6400 seedlings were arranged in a randomized complete block design with ten blocks and grown over two cultivation seasons.

Over an 80-week period, measurements included root, stem, and leaf dry weight, total leaf area, total root length, and specific root length, while leaf gas exchange and chlorophyll content (SPAD index) were monitored for 78 weeks. Species-specific differences were observed in growth rate, biomass allocation, and root traits. The seaweed-based biostimulant increased stem and total plant dry weight only at the highest (3×) dose during the first year. Significant effects on leaf gas exchange were likewise detected only at this concentration and were mainly associated with increased leaf greenness rather than reduced diffusional limitations to photosynthesis. These findings suggest that Ascophyllum-based extracts may provide limited short-term growth benefits under high application rates, potentially through improved nutrient availability. However, the requirement for elevated doses may limit their agronomic and economic feasibility, and no evidence was found for structural plant modifications associated with enhanced long-term transplant resilience.

A broader perspective is provided by the review of Cuevas Cruz & Martinez-Trinidad [46], who examined the application of biostimulants in both field and semi-controlled environments, highlighting key challenges in their use for urban tree management. In some drought-stressed urban tree species, increases in height were observed only after combined application of humic acids and seaweed extracts (50 mL, five applications), whereas biostimulants alone showed no significant effects [47]. Similarly, no beneficial effects on photosynthetic efficiency (Fv/Fm) or stress-related parameters were reported in *Quercus ilex* L., *Ilex aquifolium* L., *Sorbus aucuparia*, and *Fagus sylvatica* L. when treated with seaweed-based products such as Maxicrop Original^®^, Bioplex^®^, and Redicrop^®^ [48].

These inconsistent responses may be linked to dosage, as optimal effects have been reported only at applications up to ten times higher than recommended levels [49]. Other limiting factors include product quality [8]; application method, typically soil-based methods, which may reduce effectiveness in urban conditions [47]; and the inherently adverse characteristics of urban soils, such as high pH, compaction, low mineral content, and pollution [50]. Under such extreme conditions, seaweed-based biostimulants may not represent a reliable solution [51]. Additionally, variability in product composition, lack of standardized application protocols, and inconsistencies in the definition of biostimulants contribute to uncertainty regarding their effectiveness [8,52,53].

Nevertheless, positive outcomes have been reported in certain perennial systems. In apple (*Malus domestica*), the application of Fertiactyl Starter^®^ (1%)—containing humic and fulvic acids and glycine betaine—resulted in significantly thicker trunks after three years compared to control plants [54]. In olive trees (*Olea europaea* L.), treatments with microalgae-based products (AgriAlgae^®^; *Nannochloropsis* spp.) and seaweed extracts (Seaweed Mix^®^; *Ascophyllum nodosum* and *Laminaria* spp.) applied every 20 days under 50% irrigation increased leaf area by 26–44% and maintained stomatal conductance at levels comparable to fully irrigated plants [55].

Overall, the available evidence suggests that the effectiveness of biostimulants in perennial and urban tree systems is highly dependent on environmental conditions, plant species, product composition, dosage, and application strategy. Positive effects are more consistently observed under moderate stress conditions and when formulations are optimized for specific crops and management systems. In contrast, under severe urban soil constraints or when inappropriate doses and application methods are used, biostimulants often show limited or inconsistent efficacy. These findings highlight the need for standardized protocols and crop-specific validation under field conditions.

Microalgae-based biofertilizers have also shown promise in fruit production systems. In a field experiment conducted in a commercial hawthorn orchard (Crataegus pinnatifida) in Shanxi Province, China, Ma et al. [56] demonstrated that live microalgae applications increased fruit yield by 15.7–29.6% during a single growing season, with the highest improvement observed at medium doses applied to the roots. The study was performed under conventional fertilization in alkaline silt loam soil conditions and additionally reported increased soluble and reducing sugar content at high doses. Importantly, microalgae applications did not significantly increase greenhouse gas emissions, except for nitrous oxide under high-dose root application, while methane uptake increased by 1.5–2.3 times.

Finally, Gatti et al. [57] evaluated a commercial biostimulant (Expando), derived from *Ascophyllum nodosum* and yeast extracts, in apricot (*Prunus armeniaca* L.) cultivation. Applications at 5.0 L/ha improved fruit uniformity and harvest synchronization, while significantly enhancing the biosynthesis of bioactive compounds, including polyphenols, flavonoids, proanthocyanidins, and anthocyanins, in both pulp and peel, as confirmed by HPLC-ESI-MS/MS analysis. These metabolic enhancements led to increased antioxidant activity, particularly in the peel. Principal component analysis (PCA) further distinguished the effects of higher doses from lower treatments and controls.

Overall, while biostimulants have demonstrated clear benefits in annual crops, their application in woody and perennial species remains complex and context-dependent. Factors such as species, environmental conditions, product composition, and application strategy critically influence their effectiveness, likely due to differences in plant physiological responses, soil physicochemical properties, microbial colonization efficiency, and the stability and bioavailability of bioactive compounds under varying environmental conditions. In addition, emerging studies suggest that interactions between microalgae and associated bacterial consortia may further enhance biostimulant performance through synergistic effects on nutrient exchange, detoxification, and metabolite production [15,58,59]. The ecological mechanism behind why some microalgae perform better in association with bacteria is relevant to the phycosphere, the microscale zone surrounding algal cells in which intense exchange of metabolites and signaling molecules occurs between algae and associated bacteria. Similar to the rhizosphere in plants, the phycosphere supports interactions that can influence algal growth, nutrient acquisition, vitamin supply, stress tolerance, and metabolite production. Nonetheless, accumulating evidence supports the potential of these approaches to improve fruit quality, enhance secondary metabolism, and contribute to more sustainable and resilient agricultural systems.

### 3.3. Effects of Biostimulant Applications in Viticulture

Relatively little is known about the role of cyanobacteria-based biostimulants in viticulture. A pioneering study by Salvi et al. [60] comprehensively investigated the effects of *Arthrospira platensis* F&M-C256 extract on *Vitis vinifera* L. cv. *Pinot Nero* grown in pots under both optimal irrigation and water stress conditions. To evaluate the treatment effects, key physiological parameters of the plants, as well as qualitative and quantitative traits of the grapes, were analyzed.

The results demonstrated that treatments with *A. platensis* F&M-C256 improved leaf gas exchange in both irrigation regimes. Notably, the application of *A. platensis* F&M-C256 enabled the maintenance of stomatal opening without negatively affecting the water potential of vines subjected to drought stress.

Regarding fruit characteristics, treated vines exhibited increased berry weight compared to untreated controls under both irrigation conditions. Furthermore, under water deficit, *A. platensis* F&M-C256 improved fruit composition, indicating a positive influence on grape quality.

Overall, the findings of this study highlight a cyanobacteria-mediated physiological response under abiotic stress conditions, suggesting that *A. platensis* F&M-C256 can significantly modulate both plant performance and grape characteristics at harvest.

## 4. In Silico Validation

Building on the literature reviewed, the in silico analysis was designed to test the working hypothesis that photosynthetic microorganisms repeatedly reported in the literature as effective biostimulants or biofertilizers exhibit enriched gene representation in KEGG pathways functionally associated with plant growth promotion and stress response. Under this hypothesis, organisms with documented bioactive compound production are expected to show higher gene counts in secondary metabolism, antioxidant, and hormone signaling pathways, while organisms reported as environmentally robust are expected to show enriched representation in adaptation, stress signaling, and DNA repair pathways. The analysis was therefore conducted to evaluate the degree of agreement between literature-based biostimulant reports and predicted functional repertoires inferred from genome annotation.

A systematic search was conducted to identify fully assembled genomes of microorganisms reported in the literature to exert beneficial effects on plant growth and crop improvement. The selection was based on published evidence as well as strict genome assembly quality criteria, with only organisms for which a complete genome assembly was publicly available in curated databases being retained for analysis. Genome data were retrieved from the NCBI Genome (National Center for Biotechnology Information) [61], the European Nucleotide Archive (ENA) [62], and the Kyoto Encyclopedia of Genes and Genomes (KEGG) [62]. Only high-quality assemblies classified as complete genomes, with high completeness and minimal fragmentation, were included. Based on these criteria, a total of 19 microorganisms were selected for downstream analysis, including the cyanobacterium *Arthrospira platensis*; the green microalgae *Auxenochlorella pyrenoidosa*, *Parachlorella kessleri*, *Chlorella protothecoides*, *Chlorella sorokiniana*, *Chlorella vulgaris*, *Dunaliella salina*, *Haematococcus pluvialis*, *Monoraphidium minutum*, *Neochloris oleoabundans*, *Scenedesmus quadricauda*, *Scenedesmus vacuolatus*, and *Tetradesmus (Scenedesmus) obliquus*; the diatoms *Phaeodactylum tricornutum* and *Skeletonema costatum*; the haptophyte *Isochrysis galbana*; the brown algae *Ascophyllum nodosum* and *Saccharina japonica*; and the fungus *Trichoderma viride* (included as a non-photosynthetic reference organism, given its well-documented plant growth-promoting and biocontrol properties and its frequent co-application with microalgae in agricultural systems, as reported in Section 3.1).

Gene prediction was performed using different approaches for prokaryotic and eukaryotic organisms to account for differences in genome organization. For the prokaryotic genome, the automated annotation tool Prokka [63] was used, enabling prediction of open reading frames (ORFs), identification of rRNA and tRNA genes, functional annotation based on integrated databases, and direct generation of protein sequences in .faa format. For eukaryotic microorganisms, gene prediction was carried out using AUGUSTUS [64], which applies probabilistic models (Hidden Markov Models) for ab initio gene prediction. The default AUGUSTUS parameters were applied. Gene prediction models were selected according to taxonomic affiliation: chlorella for Chlorella species, chlamy2011 for other green microalgae, aspergillus_nidulans for fungi, and volvox for all remaining species. This process included the identification of exons, introns, and coding sequences (CDS), the generation of annotation files in .gff3 format, and the extraction of corresponding protein sequences using the script getAnnoFasta.pl, producing .aa output files. The resulting protein sequence datasets were used for downstream analysis. No manual curation or experimental validation of gene predictions was performed; analyses were based on default parameters of the respective pipelines.

Functional characterization was performed using the KEGG tools BlastKOALA and GhostKOALA [62], which assign functional annotations based on orthology. All predicted protein sequences (.aa/.faa) were submitted to these tools to obtain KEGG Orthology (KO) identifiers. These KO assignments were subsequently mapped to metabolic pathways, enabling reconstruction of metabolic networks through KEGG Mapper. The analysis generated gene-to-KO mapping tables, distributions of KO identifiers across metabolic pathways, and quantitative profiles of gene abundance per functional category. KEGG BlastKOALA and GhostKOALA assign KO identifiers based on the best ortholog match according to internal scoring criteria, and the resulting KO annotations were used as provided without additional manual filtering or re-interpretation. This quantification was used as a proxy for the metabolic potential of each organism and served as the basis for comparative analysis of pathways associated with key biological processes, including antioxidant activity, hormone production and defense-related compounds; cellular communication and stress signal transduction; DNA repair and genome stability; environmental adaptation; core cellular metabolic capacity; membrane structure, energy storage, and stress adaptation; secondary metabolite biosynthesis; stress resistance; and biodegradation and xenobiotic metabolism.

Building upon the results of the previous steps, the absolute number of genes participating in each metabolic pathway identifier associated with a given biological process was quantified. No previous filtering step was conducted, apart from the BlastKOALA and GhostKOALA results. The visualization of these quantitative data was performed using the open-source programming language R.

**Figure 1:**
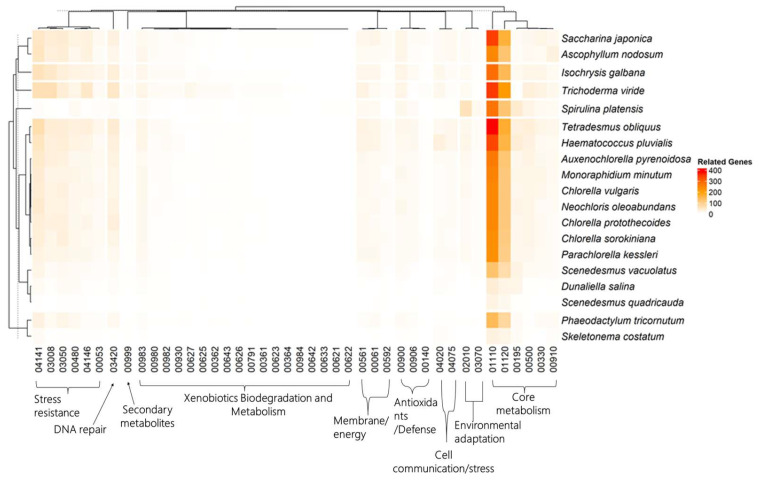
Heatmap of the absolute number of genes per organism annotated to selected KEGG pathways. Rows represent the 19 organisms analysed; columns show individual KEGG pathway map identifiers, grouped along the x-axis into nine functional categories (stress resistance, DNA repair, secondary metabolism, xenobiotic biodegradation and metabolism, membrane transport and energy, antioxidant and defense, cell communication and stress signaling, environmental adaptation, and core metabolism; see Table 2 and Table 3 for the full pathway-to-category assignment). Cell colour indicates the absolute count of genes assigned to each pathway in each organism, following a continuous gradient from white (0 genes) through pale orange and orange (intermediate counts, ~100–300 genes) to deep red (≥400 genes), as shown in the colour key on the right; white cells indicate zero or near-zero counts. Dendrograms on the top and left show hierarchical clustering of pathways and organisms, respectively (Euclidean distance). Gene models were predicted with Prokka (prokaryotes) or AUGUSTUS (eukaryotes) and functionally annotated with KEGG BlastKOALA/GhostKOALA followed by pathway mapping in KEGG Mapper. Counts are unnormalized.

**Figure 2:**
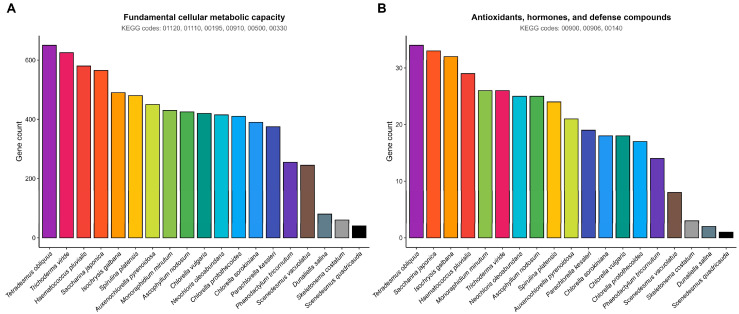
Absolute number of annotated genes per organism assigned to KEGG pathways relevant to plant growth promotion and stress resilience. (**A**) Fundamental cellular metabolic capacity, summing gene counts across six KEGG pathways: microbial metabolism in diverse environments (01120), biosynthesis of secondary metabolites (01110), photosynthesis (00195), nitrogen metabolism (00910), starch and sucrose metabolism (00500), and arginine and proline metabolism (00330). (**B**) Antioxidant, hormone, and defense-related secondary metabolism, summing gene counts across three KEGG pathways: terpenoid backbone biosynthesis (00900), carotenoid biosynthesis (00906), and steroid hormone biosynthesis (00140). Gene models were predicted from publicly available genome assemblies using Prokka (prokaryotes) or AUGUSTUS (eukaryotes) and functionally annotated through KEGG BlastKOALA/GhostKOALA; KO identifiers were mapped to pathway codes via KEGG Mapper. Counts are unnormalized.

The KEGG pathway codes investigated in this study were organized into nine functional categories. These categories were further consolidated into two major biological themes based on their functional roles: pathways associated with growth promotion (as summarized in Table 2) and pathways involved in stress response and adaptive mechanisms (as summarized in Table 3). The classification was based on the functional roles of KEGG pathways in plant–microbe systems, following established interpretations in the literature that distinguish growth-related metabolic and biosynthetic processes from stress response and adaptive signaling pathways. This grouping was used to facilitate biological interpretation and comparative analysis of functional trends across samples. The absolute gene counts for the core baseline and direct biostimulant pathways are retained in the main text (Figure 1 and Figure 2), while the specialized sub-pathway distributions are compiled in Supplementary Figures S1–S6 to facilitate a more streamlined biological interpretation.

### 4.1. General in Silico Analysis Results

To provide a comprehensive, global overview of the predicted metabolic repertoires across all 19 examined taxa, an integrated hierarchical clustering heatmap was generated for all investigated KEGG pathway categories (Figure 1). This visual profile uses absolute gene counts to show which metabolic functions are shared across these species and where their genetic differences lie.

Notably, across all taxa, a highly pronounced enrichment is observed in core metabolic capabilities, specifically within the biosynthesis of secondary metabolites (KEGG 01110) and microbial metabolism in diverse environments (KEGG 01120). This uniformly intense genetic baseline underscores a conserved metabolic engine capable of generating foundational cellular building blocks and primary precursors across diverse ecological niches.

The clustering architecture reveals distinct functional groupings that mirror the specialized ecological adaptations discussed across the literature. For instance, *T. viride* demonstrates a uniquely elevated representation in stress resistance architectures (such as glutathione metabolism and proteasome processing, KEGG 00480 and 03050) and genomic stability pathways (nucleotide excision repair, KEGG 03420). These patterns strongly correlate with established findings by Harman et al. [65] and Kubicek et al. [66], which characterize *Trichoderma* species as highly competitive opportunistic plant symbionts possessing specialized reactive oxygen species (ROS) detoxifying machineries and secondary metabolic frameworks essential for thriving in active soil microbiomes.

Similarly, the green microalga *T. obliquus* highlights an expanded repertoire in lipid remodeling pathways, including fatty acid biosynthesis (KEGG 00061) and alpha-linolenic acid metabolism (KEGG 00592), reflecting a highly flexible genomic toolkit evolved to handle intense environmental fluctuations like light and temperature variances in aquatic ecosystems.

Conversely, the heatmap captures distinct clusters of organisms that exhibit more specialized or streamlined genomic profiles. Diatoms like *Skeletonema costatum* and certain chlorophytes like *Scenedesmus quadricauda* display lower relative gene counts across several specialized categories, indicating a reliance on highly specialized or alternative physiological pathways rather than large-scale gene redundancy. Meanwhile, specialized secondary metabolite processes (KEGG 00999) show distinct concentration blocks, particularly highlighted in haptophytes like *Isochrysis galbana*. This matches extensive characterizations by Carrillo & Anchundia [67] and De Los Reyes et al. [68], which document *I. galbana* as an exceptionally rich source of complex functional lipids, antioxidants, and anti-inflammatory galactosylglycerides.

Furthermore, the cluster containing *Arthrospira platens* is exhibits sharp, isolated expansions in environmental adaptation pathways (such as ABC transporters, KEGG 02010), aligning with its characterized ecological robustness in extreme alkaline and thermophilic conditions.

Ultimately, while this global in silico profile maps out the target baseline constraints and evolutionary investments of each organism, the actual translation of these gene counts into physical biostimulant compounds remains strictly gated by real-world regulatory networks. Environmental pressures, such as nitrogen starvation or high irradiance thresholds, act as the required transcriptomic triggers to shift these organisms from genomic potential to active, plant-beneficial phenotypic expression. To dissect these critical, agronomically focused mechanisms more clearly, the core baseline metabolic capacities and targeted hormone/antioxidant defense compound pathways are examined explicitly below.

### 4.2. Genomic Repertoires Supporting Plant Growth Promotion

Core metabolic pathways—including carbohydrate, lipid, amino acid, and nitrogen metabolism, together with photosynthesis and the biosynthesis of secondary metabolites, define the metabolic baseline from which biostimulant-relevant compounds are derived. Figure 2A summarises the combined gene counts for six representative KEGG pathways in this category across the 19 organisms. Most of the examined microorganisms exhibit a high number of genes within this category, reflecting the essential role of core metabolic pathways in sustaining cellular function. The relatively uniform distribution of these genes across species suggests that fundamental metabolic functions represent a common and conserved feature among microorganisms. Notably, Tetradesmus (Scenedesmus) obliquus and Trichoderma viride display the highest gene counts, followed by Haematococcus pluvialis and Saccharina japonica, whereas Scenedesmus quadricauda and Skeletonema costatum show comparatively lower numbers of related genes.

These findings provide a genomic baseline emphasizing the importance of primary metabolic processes in growth and in the production of bioactive compounds. The presence of genes associated with nitrogen and carbohydrate metabolism indicates a strong capacity for nutrient utilization and the synthesis of metabolic products that can influence plant physiology, contributing to plant nutrition and improved growth. Furthermore, the identification of genes related to photosynthesis and secondary metabolite biosynthesis supports the ability of these organisms to produce bioactive compounds such as carotenoids, amino acids, polysaccharides, and other metabolites with potential biostimulant activity.

Crucially, while this extensive metabolic potential supports the applicability of these species in biostimulant-based agricultural practices, these absolute counts represent a genetic baseline rather than active transcription. Real-world metabolite production under field conditions requires specific environmental triggers, such as nitrogen limitation to drive lipid accumulation or light fluctuations to stimulate carotenogenesis, which dictate the transition from genomic potential to active plant growth promotion. Deeper tracking of related sub-pathways, such as specific molecular transporters and varied xenobiotic degradation profiles, are compiled in Supplementary Figures S1–S3 to maintain a concise overview of core growth-promoting architectures.

### 4.3. Genomic Drivers of Stress Resilience and Adaptive Signaling

Figure 2B narrows the analysis to three pathways directly relevant to plant-protective and hormone-like compounds: terpenoid backbone biosynthesis (00900), carotenoid biosynthesis (00906), and steroid hormone biosynthesis (00140). Gene counts here are much lower in absolute terms (1–35) but show more taxonomic structure. Tetradesmus obliquus and Saccharina japonica lead (>30 genes), followed by Isochrysis galbana and Haematococcus pluvialis—a ranking that aligns with the experimentally documented phenolic output of Saccharina and Isochrysis and the well-characterised astaxanthin production of Haematococcus (Table 1).

Importantly, a high gene count in these pathways defines a potential metabolic ceiling rather than guaranteed metabolite accumulation. Realised production depends on stress-induced regulatory cues—high irradiance, temperature shifts, or nutrient limitation—that are well documented to trigger carotenogenesis in Haematococcus and Dunaliella and phenolic accumulation in brown macroalgae. The in silico analysis presented here is therefore best interpreted as a genomic rationale for organism selection, with the empirical quantification of biostimulant-relevant metabolites under defined cultivation conditions addressed in the following sections. Additional adaptive pathways (signaling networks, membrane lipid remodeling, proteasome-mediated turnover, and nucleotide excision repair) are provided in Supplementary Figures S4–S7.

## 5. Conclusions

Among photosynthetic microorganisms, *Chlamydomonas reinhardtii* has emerged as a particularly well-characterized model for algal phytohormone production and algal–bacterial mutualism. *C. reinhardtii* has been shown to synthesize indole-3-acetic acid (IAA) and related auxin derivatives, with biosynthesis modulated by tryptophan availability and by interactions with associated bacterial partners [69]. In co-cultures with plant growth-promoting rhizobacteria such as *Methylobacterium* and *Azospirillum*, reciprocal exchange of fixed carbon, vitamins, and phytohormones has been demonstrated, with the bacterial partner often enhancing algal IAA accumulation and the algal partner supporting bacterial growth through organic carbon release [70]. Such mutualistic interactions are increasingly recognized as a key feature of the phycosphere—the diffusive boundary layer surrounding algal cells—where intense chemical signaling and nutrient exchange occur [58]. The relevance of these interactions for agriculture is twofold: first, algal-derived auxins can directly stimulate root development and lateral root formation in target crops; second, the synergistic activity of microalgal–bacterial consortia can produce a more diverse and stable pool of plant growth-promoting metabolites than either partner alone, supporting their application as combined biostimulant formulations. These observations are consistent with the present in silico findings, which identified plant hormone signal transduction (KEGG 04075) genes across several of the analyzed microalgae, and reinforce the value of considering algal–bacterial consortia rather than axenic strains in future biostimulant development. The analysis of the bar plots indicates that certain organisms consistently exhibit higher numbers of annotated genes across multiple functional categories, suggesting an expanded predicted functional repertoire across a range of biological processes. In particular, *Trichoderma viride*, *Tetradesmus* (*Scenedesmus*) *obliquus*, *Isochrysis galbana*, *Saccharina japonica*, and *Haematococcus pluvialis* show higher gene representation in categories such as core cellular metabolism, stress resistance, xenobiotic metabolism, DNA repair and stability, and secondary metabolite production. The higher abundance of annotated genes in these categories suggests potentially enhanced metabolic versatility and adaptive capacity; however, these patterns reflect annotation-based gene counts and should not be directly interpreted as differences in functional complexity without considering genome size, annotation completeness, and gene redundancy.

At the same time, differences are observed among organisms with respect to specific functional categories. For example, *Arthrospira platensis* demonstrates particularly high values in environmental adaptation. In contrast, several species of the genus *Chlorella* (such as *Chlorella vulgaris*, *Chlorella sorokiniana*, and *Chlorella protothecoides*) display relatively stable but moderate values across multiple categories, suggesting a more balanced though less diverse functional profile compared to the top-performing organisms.

Conversely, organisms such as *Skeletonema costatum* and *Scenedesmus quadricauda* exhibit lower gene counts across most of the examined biological processes, indicating a more limited functional range relative to other species. This pattern may reflect distinct ecological strategies or specialization in specific metabolic functions.

Overall, the results suggest that a small subset of organisms exhibits high gene representation across multiple metabolic and cellular pathways, whereas others display more specialized or restricted functional profiles.

Importantly, the findings of the bioinformatics analysis are in strong agreement with the literature. Several of the species exhibiting high gene representation in categories related to secondary metabolite production and antioxidant activity are also reported as important sources of bioactive compounds, including carotenoids, phenolic molecules, and phytohormones (Table 1). Furthermore, the presence of genes associated with environmental adaptation aligns with the cultivation conditions and physiological responses of microalgae [21].

The identification of genes involved in antioxidant production, secondary metabolism, and environmental adaptation further supports the potential use of certain species as sources of bioactive compounds. These results provide a foundation for the selection of promising organisms and for the optimization of cultivation conditions aimed at their exploitation in biostimulant applications and other biotechnological products.

### Limitations

Several limitations of the present work should be acknowledged. First, gene counts were used as a proxy for metabolic potential without normalization; organisms with larger genomes may show higher absolute gene counts across all categories independently of true functional specialisation. Second, gene presence does not imply expression: the identified pathways may not be active under agronomically relevant conditions, and transcriptomic or proteomic data would be required to confirm functional activity. Third, the literature review component does not include a formal PRISMA-style systematic search protocol; future iterations of this work should apply explicit inclusion and exclusion criteria to reduce selection bias. Finally, the in silico findings presented here require experimental validation under controlled cultivation conditions before any of the identified organisms can be recommended for practical biostimulant applications.

## Figures and Tables

**Table 1:** Categories of bioactive compounds identified in microalgae and cyanobacteria and their potential role in agriculture [20].

Bioactive Compounds	Biological Activity	Agricultural Function	Microalgae and Cyanobacteria Sources
Phenolic compounds	Antioxidant, antibacterial, and antifungal	Protection of crops against pathogens and various biotic and abiotic stress conditions.	*Chlorella vulgaris*, *Isochrysis* sp., *Botryococcus braunii*; *Odontella sinensis*; *Chaetoceros calcitrans*; *Phaeodactylum tricornutum*; *Isochrysis galbana*; *Tetraselmis suecica*; *Saccharina japonica*; *Neochloris oleoabundans*; *Skeletonema costatum*
Carotenoids	Antioxidant, anti-inflammatory, and anticancer	Crop enhancement, biological soil remediation and fertilization, and protection of crops against biotic and abiotic stress conditions.	*Spirulina* sp.; *Dunaliella salina*; *Chlorella pyrenoidosa*; *Haematococcus pluvialis*; *Chlorella protothecoides*; *Muriellopsis* sp.; *Chlorella zofingiensis*; *Phaeodactylum tricornutum*
Terpenoids	Antioxidant, antibacterial, and anticancer	Protection of crops against insects, bacteria, and other organisms; attraction of pollinators; and stimulation of plant growth and development.	*Pseudanabaena articulate*; *Sphaerococcus coronopifolius*; *Chondrococcus hornemanni*; *Hypnea pannosa*; *Plocamium cornutum*; *Oscillatoria perornata*; *Planktothricoids raciborskii*; *Thermosynechococcus elongate*; *Portieria hornemann*; *Pseudanabaena* sp.; *Synechocystis* sp.; *Plocamium leptophyllum*
Polysaccharides	Antioxidant, anti-inflammatory, antibacterial, anticoagulant, and anticancer	Protection of crops from biotic and abiotic stress conditions, improvement of soil quality, and stimulation of plant growth.	*Dunaliella*; *Chlorella*; *Navicula*; *Aphanothece*; *Cylindrotheca*; *Scytonema*; *Arthrospira*; *Rhodella*; *Phaeodactylum*; *Chlamydomonas*; *Porphyridium*; *Nostoc*
Free Fatty Acids	Antioxidant, antifungal, antiviral, antibiotic, and anticancer	Protection of crops against various biotic and abiotic stress conditions.	*Dunaliella*; *Spirulina*; *Chlorella*; *Porphyridium*; *Nannochloropsis*; *Anabaena*; *Scenedesmus*
Phytohormones	Chemical messengers	Crop response to stress conditions, regulation of cellular activities in crops, and stimulation of plant growth.	*Chlamydomonas*; *Chlorella*; *Protococcus*; *Scenedesmus*; *Arthrospira*; *Phormidium*

**Table 2:** KEGG codes that contribute to Plant Growth.

Action	Code	Name
Membrane transport	02010 03070	ABC transporters Bacterial secretion system
Fundamental cellular metabolic capacity	01120 01110 00195 00910 00500 00330	Microbial metabolism in diverse environments Biosynthesis of secondary metabolites Photosynthesis Nitrogen metabolism Starch and sucrose metabolism Arginine and proline metabolism
Secondary metabolites	00999	Biosynthesis of various plant secondary metabolites, including: Crocin biosynthesis, Ginsenoside biosynthesis, Saponin adjuvant biosynthesis, Cannabidiol biosynthesis, Mugineic acid biosynthesis, Pentagalloylglucose biosynthesis, Benzoxazinoid biosynthesis, Gramine biosynthesis, Coumarin biosynthesis, Furanocoumarin biosynthesis, Hordatine biosynthesis, Podophyllotoxin biosynthesis
Xenobiotics biodegradation and metabolism	00361 00362 00364 00621 00622 00623 00625 00626 00627 00633 00642 00643 00791 00930 00980 00982 00983 00984	Chlorocyclohexane and chlorobenzene degradation Benzoate degradation Fluorobenzoate degradation Dioxin degradation Xylene degradation Toluene degradation Chloroalkane and chloroalkene degradation Naphthalene degradation Aminobenzoate degradation Nitrotoluene degradation Ethylbenzene degradation Styrene degradation Atrazine degradation Caprolactam degradation Metabolism of xenobiotics by cytochrome P450 Drug metabolism—cytochrome P450 Drug metabolism—other enzymes Steroid degradation

**Table 3:** KEGG codes that contribute to Stress Response and Adaptive Mechanisms.

Action	Code	Name
Signal transduction	04020 04075	Calcium signaling pathway Plant hormone signal transduction
Membrane structure, energy storage and stress adaptation	00061 00592 00561	Fatty acid biosynthesis alpha-Linolenic acid metabolism Glycerolipid metabolism
Stress Resistance	00480 04146 04141 03050 03008 00053	Glutathione metabolism Peroxisome Protein processing in endoplasmic reticulum Proteasome Ribosome biogenesis in eukaryotes Ascorbate and aldarate metabolism
Antioxidants, hormones, and defense compounds	00900 00906 00140	Terpenoid backbone biosynthesis Carotenoid biosynthesis Steroid hormone biosynthesis
DNA repair and stability	03420	Nucleotide excision repair

## Data Availability

Data are publicly available and can be found in National Center for Biotechnology Information, the European Nucleotide Archive or the Kyoto Encyclopedia of Genes and Genomes.

## Supplementary Materials

The following supporting information can be downloaded at: https://www.mdpi.com/article/10.3390/plants15111634/s1. Figure S1. Absolute number of annotated genes per organism assigned to KEGG pathways associated with environmental adaptation (KEGG codes 02010, ABC transporters; 03070, bacterial secretion system). Gene predictions were obtained from publicly available complete genome assemblies using Prokka (prokaryotes) or AUGUSTUS (eukaryotes) with default parameters and without manual curation. Predicted protein sequences were functionally annotated through KEGG BlastKOALA and GhostKOALA, and KEGG Orthology (KO) identifiers were mapped to the corresponding pathway codes using KEGG Mapper. Values represent absolute (unnormalized) gene counts; no filtering criteria were applied beyond the default scoring thresholds of BlastKOALA/GhostKOALA; Figure S2. Absolute number of annotated genes per organism assigned to KEGG pathways associated with secondary metabolites (KEGG codes 00999, biosynthesis of various plant secondary metabolites). Gene predictions were obtained from publicly available complete genome assemblies using Prokka (prokaryotes) or AUGUSTUS (eukaryotes) with default parameters and without manual curation. Predicted protein sequences were functionally annotated through KEGG BlastKOALA and GhostKOALA, and KEGG Orthology (KO) identifiers were mapped to the corresponding pathway codes using KEGG Mapper. Values represent absolute (unnormalized) gene counts; no filtering criteria were applied beyond the default scoring thresholds of BlastKOALA/GhostKOALA; Figure S3. Absolute number of annotated genes per organism assigned to KEGG pathways associated with xenobiotics biodegration and metabolism (KEGG codes 00361, chlorocyclohexane and chlorobenzene degradation; 00362, benzoate degradation; 00364, fluorobenzoate degradation; 00621, dioxin degradation; 00622, xylene degradation; 00623, toluene degradation; 00625, chloroalkane and chloroalkene degradation; 00626, naphthalene degradation; 00627, aminobenzoate degradation; 00633, nitrotoluene degradation; 00642, ethylbenzene degradation; 00643, styrene degradation; 00791, atrazine degradation; 00930, caprolactam degradation; 00980, metabolism of xenobiotics by cytochrome P450; 00982, drug metabolism - cytochrome P450; 00983, drug metabolism - other enzymes; 00984, steroid degradation). Gene predictions were obtained from publicly available complete genome assemblies using Prokka (prokaryotes) or AUGUSTUS (eukaryotes) with default parameters and without manual curation. Predicted protein sequences were functionally annotated through KEGG BlastKOALA and GhostKOALA, and KEGG Orthology (KO) identifiers were mapped to the corresponding path-way codes using KEGG Mapper. Values represent absolute (unnormalized) gene counts; no filtering criteria were applied beyond the default scoring thresholds of BlastKOALA/GhostKOALA; Figure S4. Absolute number of annotated genes per organism assigned to KEGG pathways associated with secondary metabolites (KEGG codes 04020, calcium signaling pathways; 04075, plant hormone signal transduction). Gene predictions were obtained from publicly available complete genome assemblies using Prokka (prokaryotes) or AUGUSTUS (eukaryotes) with default parameters and without manual curation. Predicted protein sequences were functionally annotated through KEGG BlastKOALA and GhostKOALA, and KEGG Orthology (KO) identifiers were mapped to the corresponding pathway codes using KEGG Mapper. Values represent absolute (unnormalized) gene counts; no filter-ing criteria were applied beyond the default scoring thresholds of BlastKOALA/GhostKOALA; Figure S5. Absolute number of annotated genes per organism assigned to KEGG pathways associated with secondary metabolites (KEGG codes 00061, fatty acid biosynthesis; 00592, alpha-Linolenic acid metabolism; 00561, glycerolipid metabolism). Gene predictions were obtained from publicly available complete genome assemblies using Prokka (prokaryotes) or AUGUSTUS (eukaryotes) with default parameters and without manual curation. Predicted protein sequences were functionally annotated through KEGG BlastKOALA and GhostKOALA, and KEGG Orthology (KO) identifiers were mapped to the corresponding pathway codes using KEGG Mapper. Values represent absolute (unnormalized) gene counts; no filtering criteria were applied beyond the default scoring thresholds of BlastKOALA/GhostKOALA.Species such as Haematococcus pluvialis and Tetradesmus (Scenedesmus) obliquus exhibit the highest number of related genes, whereas Dunaliella salina and Skeletonema costatum show comparatively lower gene counts; Figure S6. Absolute number of annotated genes per organism assigned to KEGG pathways associated with secondary metabolites (KEGG codes 00480, glutathione metabolism; 04146, peroxisome; 04141, protein processing in endoplasmic reticulum; 03050, proteasome; 03008, ribosome biogenesis in eukaryotes; 00053, ascorbate and aldarate metabolism). Gene predictions were obtained from publicly available complete genome assemblies using Prokka (prokaryotes) or AUGUSTUS (eukaryotes) with default parameters and without manual curation. Predicted protein sequences were functionally annotated through KEGG BlastKOALA and GhostKOALA, and KEGG Orthology (KO) identifiers were mapped to the corresponding pathway codes using KEGG Mapper. Values represent absolute (unnormalized) gene counts; no filtering criteria were applied beyond the default scoring thresholds of BlastKOALA/GhostKOALA; Figure S7. Absolute number of annotated genes per organism assigned to KEGG pathways associated with secondary metabolites (KEGG codes 03420, nucleotide excision repair). Gene predictions were obtained from publicly available complete genome assemblies using Prokka (prokaryotes) or AUGUSTUS (eukaryotes) with default parameters and without manual curation. Predicted protein sequences were functionally annotated through KEGG BlastKOALA and GhostKOALA, and KEGG Orthology (KO) identifiers were mapped to the corresponding pathway codes using KEGG Mapper. Values represent absolute (unnormalized) gene counts; no filtering criteria were applied beyond the default scoring thresholds of BlastKOALA/GhostKOALA.
