# Supplementary material for: Photosynthetic Microorganisms in Plant Growth Promotion and Stress Response: Proposed Organisms with In Silico Validation
Source: Plants (Basel). 2026 May 26;15(11):1634. doi: 10.3390/plants15111634 (PMC13258984; doi:10.3390/plants15111634)
Supplement: Supplementary file 1 [file plants-15-01634-s001.zip › plants-4314775-supplementary.pdf]

Supplementary Material

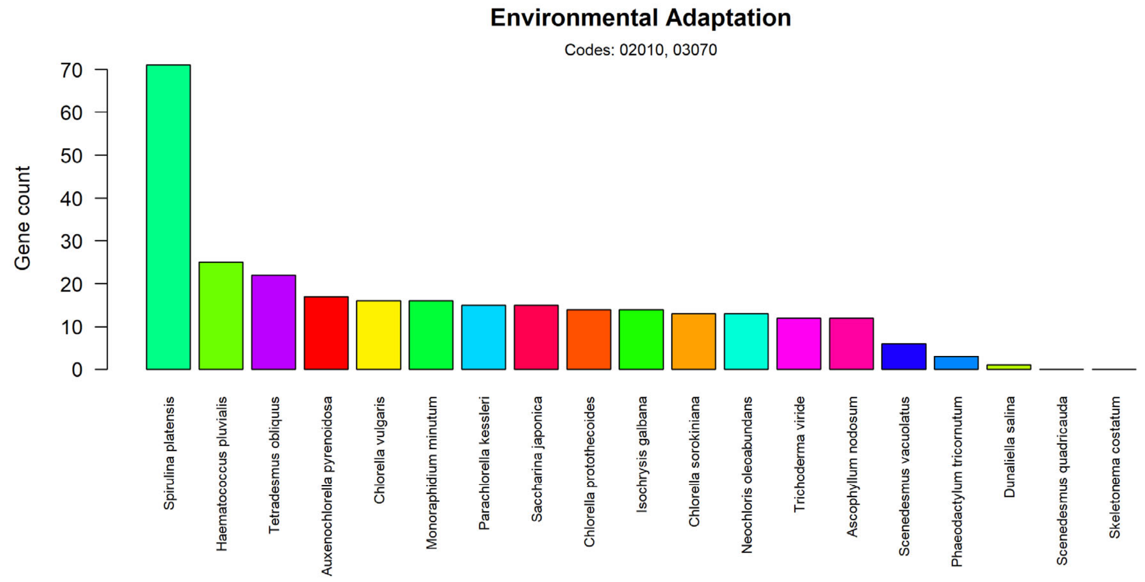

Figure S1. Absolute number of annotated genes per organism assigned to KEGG pathways associated with environmental adaptation (KEGG codes 02010, ABC transporters; 03070, bacterial secretion system). Gene predictions were obtained from publicly available complete genome assemblies using Prokka (prokaryotes) or AUGUSTUS (eukaryotes) with default parameters and without manual curation. Predicted protein sequences were functionally annotated through KEGG BlastKOALA and GhostKOALA, and KEGG Orthology (KO) identifiers were mapped to the corresponding pathway codes using KEGG Mapper. Values represent absolute (unnormalized) gene counts; no filtering criteria were applied beyond the default scoring thresholds of BlastKOALA/GhostKOALA.

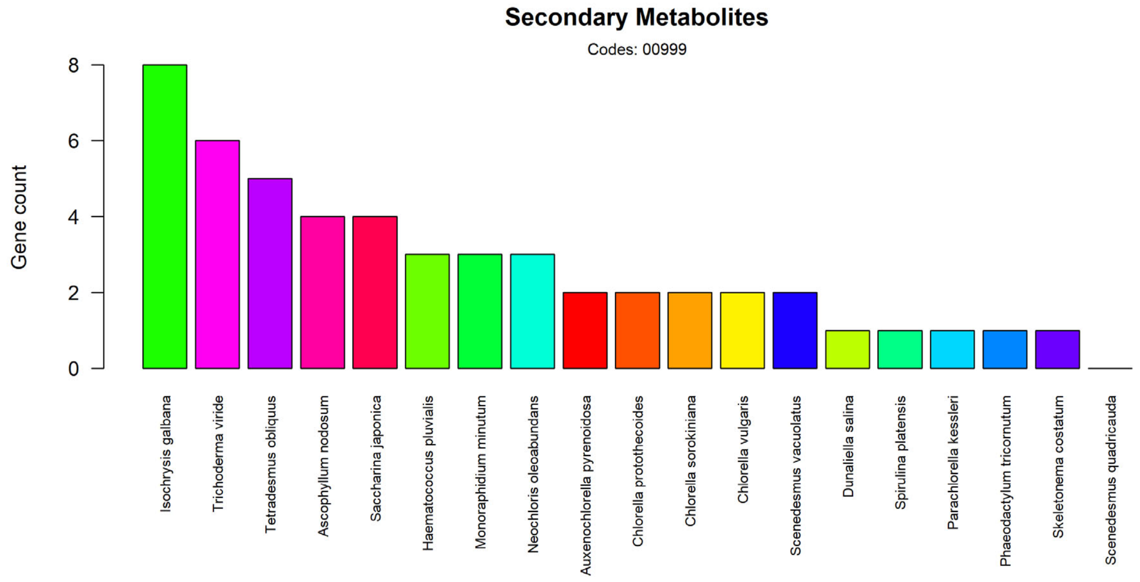

Figure S2. Absolute number of annotated genes per organism assigned to KEGG pathways associated with secondary metabolites (KEGG codes 00999, biosynthesis of various plant secondary metabolites). Gene predictions were obtained from publicly available complete genome assemblies using Prokka (prokaryotes) or AUGUSTUS (eukaryotes) with default parameters and without manual curation. Predicted protein sequences were functionally annotated through KEGG BlastKOALA and GhostKOALA, and KEGG Orthology (KO) identifiers were mapped to the corresponding pathway codes using KEGG Mapper. Values represent absolute (unnormalized) gene counts; no filtering criteria were applied beyond the default scoring thresholds of BlastKOALA/GhostKOALA.

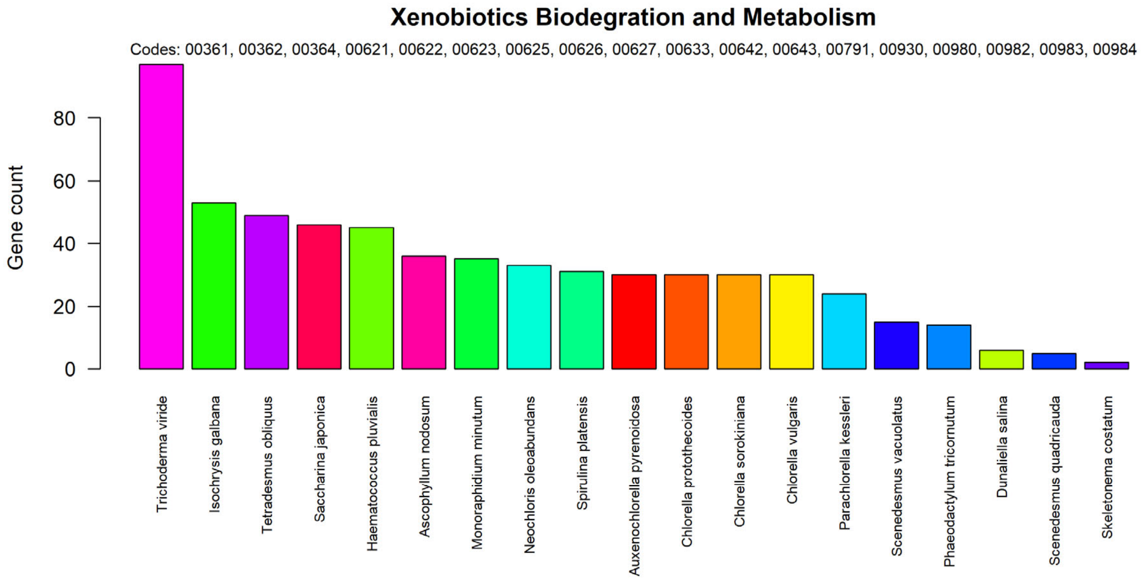

Figure S3. Absolute number of annotated genes per organism assigned to KEGG pathways associated with xenobiotics biodegration and metabolism (KEGG codes 00361, chlorocyclohexane and chlorobenzene degradation; 00362, benzoate degradation; 00364, fluorobenzoate degradation; 00621, dioxin degradation; 00622, xylene degradation; 00623, toluene degradation; 00625, chloroalkane and chloroalkene degradation; 00626, naphthalene degradation; 00627, aminobenzoate degradation; 00633, nitrotoluene degradation; 00642, ethylbenzene degradation; 00643, styrene degradation; 00791, atrazine degradation; 00930, caprolactam degradation; 00980, metabolism of xenobiotics by cytochrome P450; 00982, drug metabolism - cytochrome P450; 00983, drug metabolism - other enzymes; 00984, steroid degradation). Gene predictions were obtained from publicly available complete genome assemblies using Prokka (prokaryotes) or AUGUSTUS (eukaryotes) with default parameters and without manual curation. Predicted protein sequences were functionally annotated through KEGG BlastKOALA and GhostKOALA, and KEGG Orthology (KO) identifiers were mapped to the corresponding path-way codes using KEGG Mapper. Values represent absolute (unnormalized) gene counts; no filtering criteria were applied beyond the default scoring thresholds of BlastKOALA/GhostKOALA.

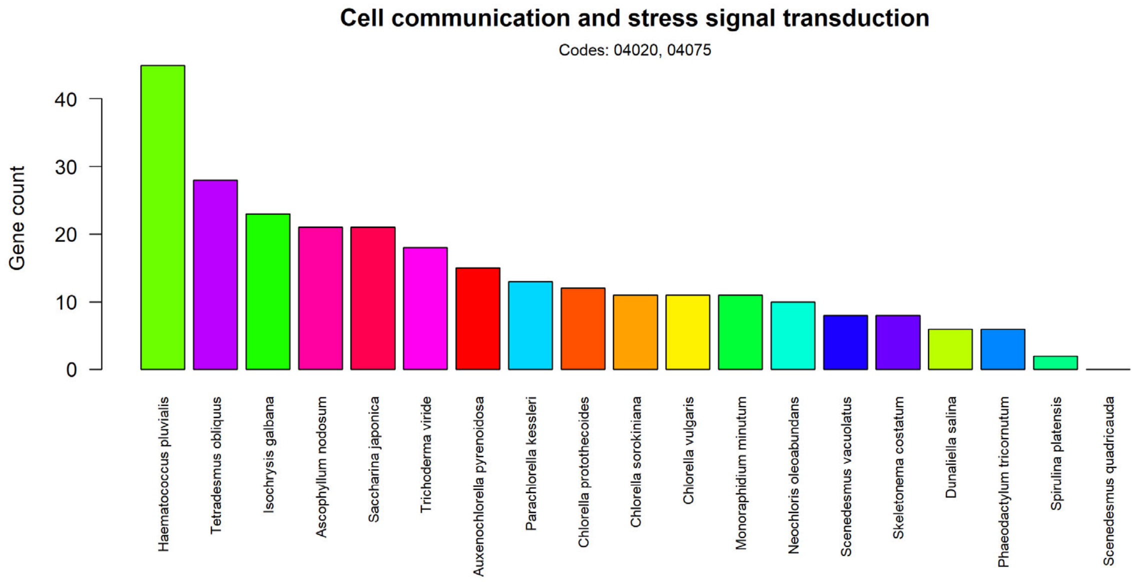

Figure S4. Absolute number of annotated genes per organism assigned to KEGG pathways associated with secondary metabolites (KEGG codes 04020, calcium signaling pathways; 04075, plant hormone signal transduction). Gene predictions were obtained from publicly available complete genome assemblies using Prokka (prokaryotes) or AUGUSTUS (eukaryotes) with default parameters and without manual curation. Predicted protein sequences were functionally annotated through KEGG BlastKOALA and GhostKOALA, and KEGG Orthology (KO) identifiers were mapped to the corresponding pathway codes using KEGG Mapper. Values represent absolute (unnormalized) gene counts; no filter-ing criteria were applied beyond the default scoring thresholds of BlastKOALA/GhostKOALA.

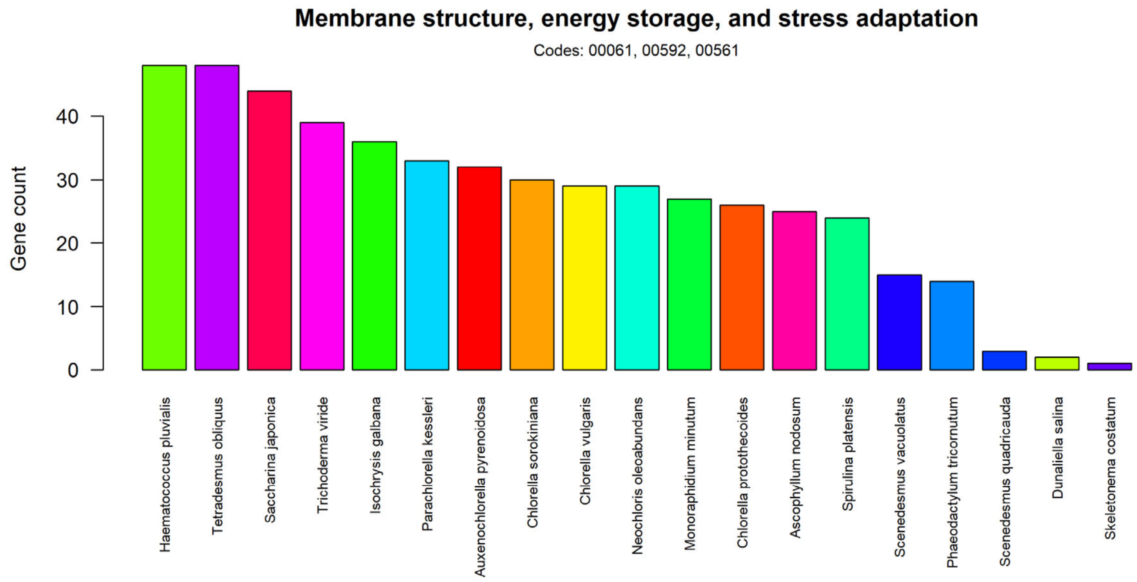

Figure S5. Absolute number of annotated genes per organism assigned to KEGG pathways associated with secondary metabolites (KEGG codes 00061, fatty acid biosynthesis; 00592, alpha-Linolenic acid metabolism; 00561, glycerolipid metabolism). Gene predictions were obtained from publicly available

complete genome assemblies using Prokka (prokaryotes) or AUGUSTUS (eukaryotes) with default parameters and without manual curation. Predicted protein sequences were functionally annotated through KEGG BlastKOALA and GhostKOALA, and KEGG Orthology (KO) identifiers were mapped to the corresponding pathway codes using KEGG Mapper. Values represent absolute (unnormalized) gene counts; no filtering criteria were applied beyond the default scoring thresholds of BlastKOALA/GhostKOALA.Species such as *Haematococcus pluvialis* and *Tetradesmus (Scenedesmus) obliquus* exhibit the highest number of related genes, whereas *Dunaliella salina* and *Skeletonema costatum* show comparatively lower gene counts.

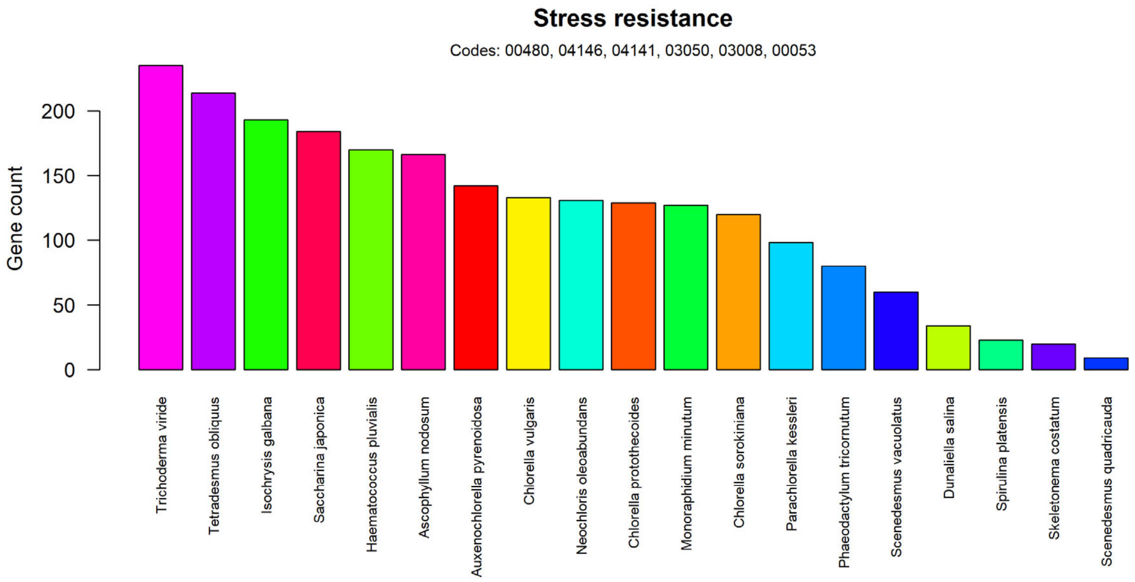

Figure S6. Absolute number of annotated genes per organism assigned to KEGG pathways associated with secondary metabolites (KEGG codes 00480, glutathione metabolism; 04146, peroxisome; 04141, protein processing in endoplasmic reticulum; 03050, proteasome; 03008, ribosome biogenesis in eukaryotes; 00053, ascorbate and aldarate metabolism). Gene predictions were obtained from publicly available complete genome assemblies using Prokka (prokaryotes) or AUGUSTUS (eukaryotes) with default parameters and without manual curation. Predicted protein sequences were functionally annotated through KEGG BlastKOALA and GhostKOALA, and KEGG Orthology (KO) identifiers were mapped to the corresponding pathway codes using KEGG Mapper. Values represent absolute (unnormalized) gene counts; no filtering criteria were applied beyond the default scoring thresholds of BlastKOALA/GhostKOALA.

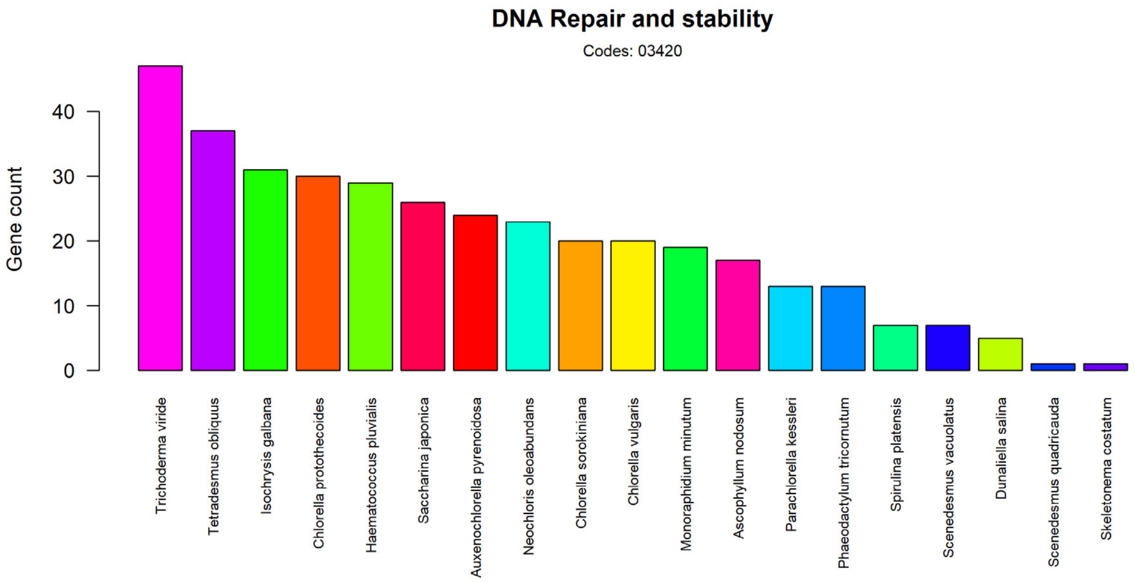

FigureS7. Absolute number of annotated genes per organism assigned to KEGG pathways associated with secondary metabolites (KEGG codes 03420, nucleotide excision repair). Gene predictions were obtained from publicly available complete genome assemblies using Prokka (prokaryotes) or AUGUSTUS (eukaryotes) with default parameters and without manual curation. Predicted protein sequences were functionally annotated through KEGG BlastKOALA and GhostKOALA, and KEGG Orthology (KO) identifiers were mapped to the corresponding pathway codes using KEGG Mapper. Values represent absolute (unnormalized) gene counts; no filtering criteria were applied beyond the default scoring thresholds of BlastKOALA/GhostKOALA.
